# Antifungal Activities of Pure and ZnO-Encapsulated Essential Oil of *Zataria multiflora* on *Alternaria solani* as the Pathogenic Agent of Tomato Early Blight Disease

**DOI:** 10.3389/fpls.2022.932475

**Published:** 2022-07-05

**Authors:** Arezou Akhtari, Mahdi Davari, Aziz Habibi-Yangjeh, Asgar Ebadollahi, Solmaz Feizpour

**Affiliations:** ^1^Department of Plant Protection, Faculty of Agriculture and Natural Resources, University of Mohaghegh Ardabili, Ardabil, Iran; ^2^Department of Chemistry, Faculty of Science, University of Mohaghegh Ardabili, Ardabil, Iran; ^3^Department of Plant Sciences, Moghan College of Agriculture and Natural Resources, University of Mohaghegh Ardabili, Ardabil, Iran

**Keywords:** *Alternaria solani*, *Zataria multiflora*, ZnO, essential oil nanocapsules, plant disease management

## Abstract

The utilization of plant essential oils (EOs) and nanomaterials due to their safety compared with synthetic chemicals has been considered in the management of plant diseases. In this study, the inhibitory effects of *Zataria multiflora*, *Nepeta haussknechtii*, *Artemisia sieberi*, and *Citrus aurantifolia* EOs in pure and Zinc Oxide (ZnO) nanocapsulated formulations were evaluated on the mycelial growth of *Alternaria solani* to find a suitable alternative for synthetic chemicals. The crystal structure and morphological properties of the fabricated nanomaterials were assessed *via* X-ray diffraction (XRD) and scanning electron microscope (SEM) analyses. The textural features of the prepared nanoparticles were investigated with Brunauer–Emmett–Teller (BET) analysis, and the presence of elements in the samples was studied with energy-dispersive X-ray (EDX) technique. The mycelial growth inhibitory (MGI) was performed in the laboratory by mixing with potato dextrose agar (PDA) medium at concentrations of 100, 300, 600, 1,000, 1,500, and 2,000 ppm. Based on the results, major differences were monitored between different concentrations. At the highest studied concentration, the inhibition of *Z. multiflora* EO was 100%, which was 43.20, 42.37, and 21.19% for *N. haussknechtii*, *A. sieberi*, and *C. aurantifolia*, respectively, and the inhibition of their nanocapsules was 100, 51.32, 55.23, and 26.58%, respectively. In the greenhouse study, *Z. multiflora* EO and its nanocapsule (ZnO-ZmEO) were compared with the ZnO and chlorothalonil fungicide based on the highest inhibitory of *Z. multiflora in vitro*. The highest antifungal effect was related to the ZnO-ZmEO by 53.33%. Therefore, the ZnO-ZmEO formulation can be recommended as a biofungicide for managing and controlling tomato early blight disease after further research.

## Introduction

Tomatoes are one of the most vital vegetable crops in the human diet worldwide due to the presence of carotene (a precursor to vitamin A), a variety of vitamins, including vitamin C, ascorbic acid, and lycopene, which is one of the strongest types of natural antioxidants. Their importance to human health has been proved (Rao and Agarwal, 2000; Jacob et al., 2008). Early blight is a typical tomato fungal disease caused by *Alternaria solani* Sorauer, which is usually identified by the occurrence of brown to dark brown necrotic lesions, having concentric rings and drying and falling leaves prematurely, and causes quantitative and qualitative damage to the product, if not adequately managed (Chohan et al., 2015). The development of elliptical to dark brown to black cankers on the stem is another obvious symptom of this disease (El-Nagar et al., 2020).

The most common method of plant diseases management is the utilization of synthetic chemicals that are often degraded slowly in nature, and since tomatoes are commonly eaten fresh, the residual risks of these chemicals on this product are of particular importance to human health (Raza et al., 2016). Also, the application of fungicides for controlling fungal plant diseases has led to the emergence of resistant races of fungal pathogens and has resulted in more health hazards such as increasing the risk of cancer and environmental pollution (Narayanasamy, 2002; Gupta and Dikshit, 2010). Nowadays, the application of essential oils (EOs) in plant disease management has been recommended as a useful and safe approach and has been considered to minimize the side effects of synthetic chemicals, and the antifungal and antimicrobial effects of several plant compounds on some plant pathogens have been proved (Beyki and Alizadeh, 2006; Davari and Ezazi, 2017; Raveau et al., 2020). In addition to being able to control plant diseases, these compounds prevent the destruction of ecological balance (Hasanzadeh, 2005; Kizil and Uyar, 2006). In spite of having many advantages, the disadvantages of EOs are high cost, physical and chemical instability, low water solubility, decomposition with oxygen, and high vapor pressure. These compounds also have organoleptic effects and cause taste and odor in products, which are not to the satisfaction of the consumers (Donsì et al., 2011). One of the best approaches to resolve the difficulties related to the EOs’ instability and volatility is nanoencapsulation (Pavoni et al., 2020). Nanomaterials protect EOs against interactions with other compounds and increase the stability of volatile compounds and antimicrobial properties through cell adsorption (Donsì et al., 2011). Also, nanoparticles can lead to effective delivery and valid release in soil-borne disease control and management (Kutawa et al., 2021).

Nanomaterials and EOs have been widely investigated for their antifungal potential in numerous reports. In particular, EOs of various medicinal plants [*Tagetes erecta* L., *Zataria multiflora* Boiss, *Melaleuca alternifolia* (Maiden & Betche) Cheel] have shown beneficial antifungal effects against *A. solani* (Hendges et al., 2021; Karimi and Meiners, 2021; Mugao et al., 2021). The antifungal properties of Fe_3_O_4_/ZnO/AgBr nanocomposites against *Fusarium oxysporum* Schltdl. and *F. graminearum* Schwabe were proved (Hoseinzadeh et al., 2016). The antifungal characteristics of TiO_2_/AgBr were assessed through the inhibition of *Sclerotinia sclerotiorum* (Lib.) de Bary, *Botrytis cinerea* Pers., and *F. graminearum*, and the findings revealed that the TiO_2_/AgBr materials have better antifungal activities on *F. graminearum* spores than the pure TiO_2_ (Habibi-Yangjeh et al., 2021). Moreover, zinc oxide nanoparticles (ZnO NPs) have been employed in many antifungal assessments, and they presented the important potential to diminish the growth of phytopathogenic fungi (Kalia et al., 2020). The mechanism of the inhibitory effect of ZnO NPs on microorganisms has not been understood. Various studies have reported that the integration of ZnO NPs increased carbohydrate and nucleic acid contents of fungi. The increase in nucleic acid may be due to stress response of fungal hyphae. The increase in carbohydrates may be due to the self-protecting mechanism against the ZnO NPs (He et al., 2011). Also, the antifungal mechanisms provided by metal NPs can be related to a disturbance of fungal cell membrane integrity, and with the generation of reactive oxygen species (ROS), which enhances the membrane disintegration process (Kumari et al., 2019). The antifungal properties of encapsulated *Thymus daenensis* L. and *Anethum graveolens* L. EOs in copper nanomaterials against the phytopathogenic agent *Colletotrichum nymphaeae* (Pass.) Aa showed that thyme and dill EOs’ encapsulation with copper nanomaterials led to an effective 90% reduction in the mycelium growth (Weisany et al., 2019). Therefore, in this study, the antifungal effects of the EOs extracted from some Iranian medicinal plants, including *Z. multiflora*, *N. haussknechtii* Bornm., *Artemisia sieberi* L., *Citrus aurantifolia* Swingle, and their nanocapsulated formulations by ZnO were evaluated to find out the fungus causal agent of tomato early blight disease.

## Materials and Methods

### Preparation of Pathogenic Fungus

After visiting the tomato fields in Bilehsavar, Parsabad, and Ardabil (Samian region) cities of Ardabil province, the samples from leaves showing signs of early blight disease were collected during the summer of 2020. After surface sterilization by dipping in 0.5% sodium hypochlorite, rinsing three times in distilled water, and drying in a sterile filter paper, the samples were plated on potato dextrose agar (PDA) medium. *A. solani* was detected by colony color, mycelial growth appearance, and microscopic morphological characteristics based on the identification key (Yadav, 2015).

### Preparation and Analysis of Essential Oils

Aerial parts of *N. haussknechtii* (Pune-sa) were obtained from the Heiran region of Ardabil, and *A. sieberi* (White wormwood) were collected from around Shurabil lake of Ardabil during the spring and summer of 2020. The plants were identified using Iranian flora sources, including flora Iranica (Rechinger, 1963–2015) and Iranian flora (Assadi, 1987–2018). Then, the dried plant samples were ground and 50 g of each sample was placed in a Clevenger-type instrument and the related EO was achieved by steam distillation method within 3 h. The as-obtained EOs were placed in dark vials at 4°C for further experiments and bioassays. *C. aurantifolia* (lime) and *Z. multiflora* (Shirazi thyme) EOs were purchased from Tabib Daroo Company.

To determine the chemical constituents of EOs, a gas chromatograph (HP-7890B) linked to a mass spectrometer (Agilent-MSD5975C) was used. Helium (99.99%) was applied as a carrier gas at a flow rate of 1 ml/min. The temperature of the injector was maintained at 250°C and a determined amount of EO was introduced. The EOs’ components were determined by employing various factors like retention time, comparing the mass spectra with those of standards such as Wiley 7n 0.1 (Wiley, NY, United States) and NIST (Standard Reference Data, Gaithersburg) (Adams, 2007; Enayati et al., 2021).

### Preparation of ZnO and Nanoencapsulation of Essential Oils With ZnO

For the nanoencapsulation of EOs with ZnO, 1.8 g Zn (NO_3_)_2_.6H_2_O (Loba Chemie, India) was dissolved in 90 ml of water. Then, the EO (1 ml) mixed with ethanol (9 ml) was appended to the obtained solution and stirred for 60 min. Next, the pH of the solution was regulated to 10 by NaOH (1 M). The obtained precipitate was ultrasonicated for 120 min (Bandelin model HD 3100). The obtained material was rinsed with H_2_O and ethanol, and the sample was dried (Kedia and Kishore Dobey, 2018).

For fabrication of pure ZnO, 1.8 g Zn (NO_3_)_2_.6H_2_O was introduced into deionized H_2_O (100 ml) and stirred for 30 min. Thereafter, the pH of solution was regulated to 10 by NaOH (1 M). The solution was ultrasonicated for 2 h. The precipitate was rinsed and dried (Pirhashemi and Habibi-Yangjeh, 2017).

### Characterization

The N_2_ adsorption–desorption isotherms were conducted using BELSORP mini II instrument. To evaluate the morphology of materials, scanning electron microscope (SEM) (LEO 1430VP) instrument was utilized. The thermal properties of samples were characterized by thermogravimetric analysis (TGA) (HORIBA model, Japan). The Fourier transform infrared (FT-IR) spectra were collected *via* a Perkin Elmer Spectrum RX I. The phase information of the samples was determined using a Philips Xpert diffractometer. X-ray energy-dispersive analysis (Rontec GmbH, Germany) was conducted to determine the chemical structure of the materials. A density meter (Anton paar DMA 4500M) was applied to measure the density of compounds. To explore the size of particles, a dynamic light scattering, HORIBA instrument was employed (Enayati et al., 2021).

### *In vitro* Antifungal Study

Antifungal effect was performed *in vitro* on *A. solani* by adding various concentrations of EOs to PDA culture media. The EOs’ emulsion was obtained *via* Tween 80 (0.05% v/v) and mixed with PDA at 40–45°C to achieve concentrations of 100, 300, 600, 1,000, 1,500, and 2,000 μl/L for each EO. Thereafter, the mixture was introduced into 9 cm Petri dishes and coagulated. A mycelial disc (5 mm) of 7-day-old cultures of *A. solani* was located on the Petri dishes, and in control ones, only Tween 80 was added. The experiment was performed in three replications. Inoculated plates were sealed to hinder evaporation of each EOs and incubated at 25°C. After 48 h, the measurement of the mycelial colonies growth rate was started and continued daily until the fungus grown in the control plates filled them. The inhibitory percentage of various concentrations of EOs and nanocapsules of EOs was calculated *via* Abbott’s formula: IP = (C − T/C) × 100, where IP is the percentage of inhibition, C and T are the mean diameter of fungal colony in control and treated plates, respectively. For investigating the fungicide or fungistatic activity of EOs and EO nanocapsules, mycelial disks with no growth were added to PDA media without treatment and tested after 7 days (Davari and Ezazi, 2017; Enayati et al., 2021).

### *In vivo* Antifungal Study

Tomato seeds (*Solanum lycopericum* L.) cultivar Super Strain B were used. The inoculum was prepared by culturing *A. solani* on PDA culture medium (27°C) for 15 days. Then, after adding sterile distilled H_2_O (10 ml) to different plates, the obtained colonies were precisely scraped with a sterile needle. The obtained conidial fungal suspension was adjusted to 5 × 10^6^ spores/ml and employed for the inoculation. The plants were covered with polyethylenic bags for 2 days to provide high humidity conditions after inoculation. Thereafter, the bags were removed and plants were maintained under greenhouse conditions (Nashwa and Abo-Elyousr, 2013). After the onset of early symptoms (about 10 days), the most effective concentrations of *Z. multiflora*, ZnO-ZmEO (ZnO-*Z. multiflora* EO), and ZnO in five repetitions and three concentrations were evaluated under greenhouse conditions. To evaluate the effect of these materials, a healthy control treatment (without infection) and an infected control treatment (by phytopathogenic fungus only) were considered. The study was performed in a completely randomized design with five replications. The intensity of the disease was recorded 2 weeks after inoculation following the score chart from 0 to 9 scales according to Latha et al. (2009): 0 = healthy; 1 = 1–5%; 3 = 6–10%; 5 = 11–25%; 7 = 26–50%; and 9 = >51% of the leaf area infected. Percent disease index (PDI) was computed based on McKinney (1923): PDI = (Σ of ratings of infected leaves observed/no. of leaves observed × maximum disease grade) × 100.

### Statistical Analysis

The analyses were done using SPSS v. 24 (IBM, Chicago, IL, United States). Data were subjected to one-way analysis of variance (ANOVA), and the comparison of means for growth inhibition percentages of *A. solani* was done using Tukey’s *post hoc* test (*p* < 0.05). The software was also used to calculate IP_50_ (concentration required to inhibit 50% mycelial growth) along with concentration-dependent regression line details and χ^2^ test to evaluate data heterogeneity.

## Results

### Chemical Composition of Essential Oils

The analysis of *Z. multiflora* EO identified thymol (47.08%), γ-terpinene (17.27%), *p*-cymene (11.95%), and carvacrol (11.63%) as the main compounds. Nepetalactone (17.78%), 1,8-cineole (13.6%), and 2-methylbicyclo[3.3.1]nonane (10.05%) were the major components of the *N. haussknechtii* EO. β-Thujone (20.01%), 1,8-cineole (16.25%), and camphor (12.52%) were recorded as the most abundant components in *A. sieberi* EO. Furthermore, limonene (32.26%), α-terpineol (12.49%), and γ-terpinene (9.31%) were the main compounds in *C. aurantifolia* EO (Table 1).

**TABLE 1:** Type and percentages of main components identified in the essential oil of *Z. multiflora*, *N. haussknechtii*, *A. sieberi*, and *C. aurantifolia*.

Essential oil	Compounds	Retention time	Percentage
*Z. multiflora*	α-Pinene	5.179	1.14
	p-Cymene	7.176	11.95
	γ-Terpinene	7.639	17.27
	Linalool	8.561	7.67
	Terpinen-4-ol	10.621	2.1
	Thymol	14.861	47.08
	Carvacrol	15.782	11.63
*N. haussknechtii*	β-Pinene	6.467	3.09
	1,8-Cineole	7.439	13.6
	Allo-ocimene	9.391	1.31
	Creosol	11.548	1.08
	Nepetalactone	15.342	17.78
	1-Methyl-1-(2-methyl-2-propenyl)cyclopentane	15.41	4.35
	3-(Hydroxymethyl)-6,7-dihydro-5H-1-benzofuran-4-one	15.53	0.58
	Chrysanthemal	15.937	7.8
	Epinepetalactone	15.96	1.33
	Caryophyllene	16.337	64
	Germacrene D	17.59	1.01
	Bicyclogermacrene	17.899	0.58
	(1-Ethyl-2-methylpropyl)methylamine	17.968	0.53
	Sabinaketone	18.237	1.36
	1S-Calamenene	18.431	2.77
	(E)-2-(2-methylpropylidene)-4-methylcyclohexanone	18.431	1.44
	1-Hexylcyclohexene	18.615	0.63
	Cyclohexene,3-(1-methylpropyl)	18.712	0.67
	1-Butylcyclohexene	18.866	1.09
	Spiro(5,6)dodecane	18.981	1.38
	2-Methylbicyclo[3.3.1]nonane	19.084	10.05
	5-Methylspiro[3.4]octan-1-one	19.479	0.59
	Caryophyllene oxide	19.524	1.73
	Spathulenol	19.713	1.95
	Veridiflorol	19.948	0.74
	(4S,8R)-8-epi-b-bisabolol	21.332	1.13
*A. sieberi*	Camphene	5.928	2.91
	Sabinene	6.426	0.84
	2,3-Dehydro-1,8-cineole	6.747	0.51
	α-Terpinene	7.199	1.25
	O-Cymene	7.359	0.74
	1,8-Cineole	7.536	16.25
	γ-Terpinene	8.103	1.62
	Terpinolene	8.749	0.73
	Artemesia alcohol	8.789	0.53
	β-Thujone	9.402	20.01
	p-Menth-2-en-1-ol	9.676	0.65
	1,6-Dimethylhepta-1,3,5-triene	9.751	1.93
	Camphor	10.443	12.52
	6,6-Dimethyl-2-methylene-bicyclo[2.2.1]heptan-3-one	10.792	1.16
	Methyl bornyl ether	10.964	3.93
	Terpinen-4-ol	11.313	4.56
	α-Terpineol	11.605	0.78
	Myrtenol	11.805	1.54
	Piperitol isomer I	12.108	0.53
	2-Methyl-3-phenylpropanal	13.19	0.62
	d-Carvone	13.361	0.5
	2-(2-Methyl-1-propenyl)-1-vinylcyclobutanol	13.739	1.14
	Verbenyl acetate	14.048	0.96
	Bornyl acetate	15.078	1.26
	Sabinyl acetate	15.37	1.97
	α-Fenchene	15.639	1.02
	Isoterpinolene	17.458	0.55
	2-Ethylidene-6-methyl-3,5-heptadienal	19.123	2.93
	Caryophyllene	19.839	0.53
	Germacrene D	21.824	5.33
	Bicyclogermacrene	22.202	1.35
	Spathulenol	24.41	1.23
	Davanone	24.702	3.63
*C. aurantifolia*	α-Pinene	5.551	3.06
	β-Pinene	6.237	8.83
	β-Myrcene	6.569	1.53
	Limonene	7.33	32.26
	β-Ocimene	7.628	0.94
	g-Terpinene	7.679	9.31
	a-Terpinolene	8.263	4.23
	4-Carene	8.343	0.69
	Linalool	8.555	2.92
	d-Fenchyl alcohol	9.013	0.76
	p-Menthan-1-ol	9.722	0.58
	Borneol	10.329	0.8
	(-)-Terpinen-4-ol	10.517	2.71
	α-Terpineol	11.233	12.49
	β-Citronellene	13.012	0.76
	Citral	13.63	0.57
	α-Terpinene	16.239	0.55
	Geranyl acetate	17.269	1.03
	β-Elemene	18.208	0.53
	Caryophyllene	19.152	2.03
	α-Bergamotene	19.678	2.32
	α-Farnesene	21.898	4.21

*The compounds with percentage less than 0.5% were not mentioned.*

### Characterization of the Materials

The density of *Z. multiflora*, *N. haussknechtii*, *A. sieberi*, and *C. aurantifolia* EOs was calculated as 0.92, 1.01, 0.92, and 0.86 g/ml, respectively.

**FIGURE 1:**
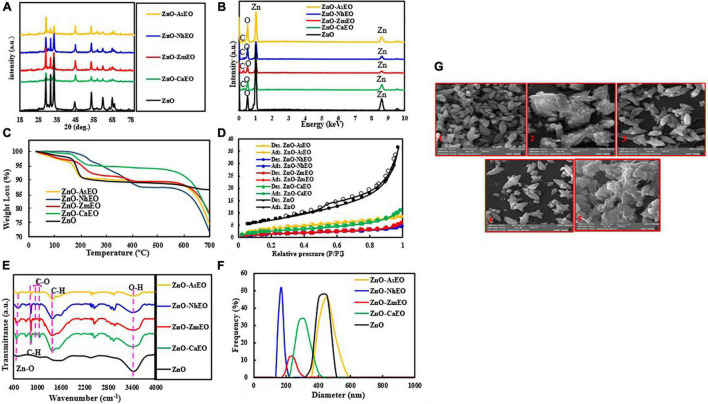
**(A)** XRD patterns, **(B)** EDS spectra, **(C)** TGA diagrams, **(D)** BET plots, **(E)** FT-IR spectra, and **(F)** DLS analyses of the ZnO and ZnO-EO samples, and **(G)** SEM images of the prepared samples: 1, ZnO; 2, ZnO-CaEO; 3, ZnO-ZmEO; 4, ZnO-NhEO; and 5, ZnO-AsEO.

The X-ray diffraction (XRD) patterns were recorded to identify the crystalline nature of the nanomaterials, and the obtained patterns are demonstrated in Figure 1A. As shown, the diffraction peaks correspond to the hexagonal crystalline structure of ZnO and are attributed to the JCPDS reference number 080-0075, confirming that encapsulations with the EOs have not changed the phase structure of ZnO particles (Beshkar et al., 2022). The absence of other diffraction peaks reveals the high purity of the fabricated materials.

The elemental characterization of the ZnO and ZnO-EO was studied by energy-dispersive X-ray spectroscopy (EDS) analysis, and the related spectra are seen in Figure 1B. The Zn and O peaks were displayed in the EDS of the ZnO NPs. The EDS spectra of the ZnO-ZmEO, ZnO-NhEO (ZnO-*N. haussknechtii* EO), ZnO-AsEO (ZnO-*A. sieberi* EO), and ZnO-CaEO (ZnO-*C. aurantifolia* EO) samples (*Z. multiflora*, *N. haussknechtii*, *A. sieberi*, and *C. aurantifolia* nanocapsules, respectively) demonstrate the presence of Zn, C, and O elements. The obtained spectra manifested that the EO molecules are successfully merged with ZnO particles.

Thermogravimetric analysis is an efficient method to evaluate the thermal stability of the prepared materials. As manifested in Figure 1C, the ZnO-EO samples lose more weight than the pure ZnO sample. For the pure ZnO, the weight loss is about 13.4%, which is because of the loss of adsorbed water molecules. Weight loss in biologically synthesized ZnO samples is owing to the loss of EOs as well as loss of adsorbed water molecules. From these analyses, the contents of *Z. multiflora*, *C. aurantifolia*, *A. sieberi*, and *N. haussknechtii* in the ZnO-EO samples are approximately 21.8, 22.5, 25.6, and 28.3%, respectively.

The Brunauer–Emmett–Teller (BET) test has been examined to get information on the surface area of the materials. The BET curves of ZnO, ZnO-ZmEO, ZnO-NhEO, ZnO-AsEO, and ZnO-CaEO samples are illustrated in Figure 1D. The N_2_ adsorption–desorption curves of the materials are consistent with the type II isotherm and mesoporous structure. Specific surface areas for ZnO, ZnO-ZmEO, ZnO-CaEO, ZnO-NhEO, and ZnO-AsEO samples were 25.5, 5.23, 9.83, 5.77, and 14.6 m^2^/g, respectively. The textural characteristics of the materials were estimated by BET and Barrett–Joyner–Halenda models and the outcomes tabulated (see Table 2).

**TABLE 2:** Textural characteristics of the ZnO and ZnO-EOs materials.

Sample	Surface area (m^2^g^–1^)	Mean pore diameter (nm)	Total pore volume (cm^3^g^–1^)
ZnO	25.5	8.91	0.0567
ZnO-CaEO	9.83	6.95	0.0170
ZnO-ZmEO	5.23	6.70	0.0087
ZnO-NhEO	5.77	4.63	0.0066
ZnO-AsEO	14.6	3.48	0.0127

To clarify the functional groups of the materials, FT-IR analysis was performed and the resulting spectra are shown in Figure 1E. In the spectra of ZnO and ZnO-EO materials, the peak at 3,400 cm^–1^ is assigned to the adsorbed H_2_O on the material’s surface. Moreover, the absorption band centered at 560 cm^–1^ belongs to the stretching vibration of Zn–O groups (Xue et al., 2022). For the ZnO-EO materials, the absorption bands at 840 and 1,380 cm^–1^ are ascribed to the C–H bending; besides, the bands at 960 and 1,052 cm^–1^ are related to the stretching vibration of C–O groups (Ertani et al., 2018; Luque et al., 2018; Wan Mat Khalir et al., 2020).

The particle size distribution of the ZnO, ZnO-ZmEO, ZnO-CaEO, ZnO-NhEO, and ZnO-AsEO materials was obtained by dynamic light scattering technique, and the results are given in Figure 1F and Table 3.

**TABLE 3:** DLS size distribution data of ZnO and ZnO-Eos.

Sample	Mean (nm)	SD (nm)	Mode (nm)	*Z*-average (nm)	PI
ZnO	398.6	29.4	402.8	5246.3	1.382
ZnO-CaEO	284.8	35.6	288.4	2647.0	1.294
ZnO-ZmEO	225.5	22.1	252.5	2873.2	7.310
ZnO-NhEO	157.4	12.9	157.0	5664.2	6.384
ZnO-AsEO	425.2	40.3	421.1	3057.4	1.865

To evaluate the morphology of the materials, SEM images were provided. Figure 1G manifested the SEM images of the ZnO, ZnO-ZmEO, ZnO-CaEO, ZnO-NhEO, and ZnO-AsEO samples. As observed in these images, the ZnO (Figure 1G1), ZnO-ZmEO (Figure 1G3), and ZnO-NhEO (Figure 1G4) samples have spindle-shaped structures.

### *In vitro* Antifungal Study

**FIGURE 2:**
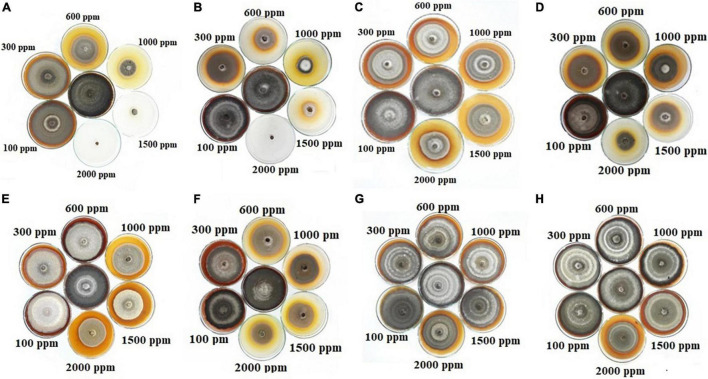
Antifungal activity of EOs and nanocapsules on the growth rate of *A. solani* at various concentrations of 0–2,000 ppm: **(A)** *Z. multiflora*, **(B)** ZnO-ZmEO, **(C)** *N. haussknechtii*, **(D)** ZnO-NhEO, **(E)** *A. sieberi*, **(F)** ZnO-AsEO, **(G)** *C. aurantifolia*, and **(H)** ZnO-CaEO.

**FIGURE 3:**
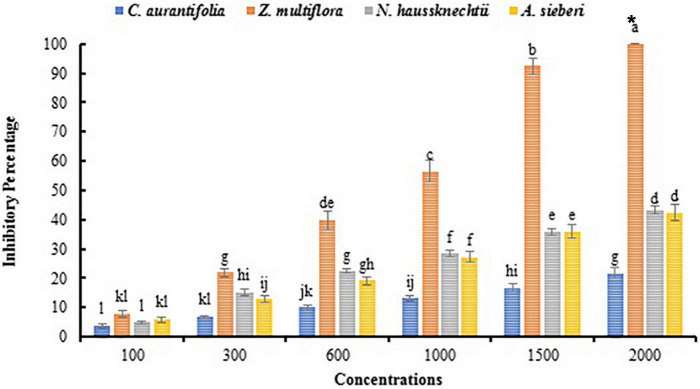
Comparison of mean inhibitory percentage of *A. solani* mycelial growth with EOs *in vitro*. The symbol “*” indicates the fungistatic property.

**FIGURE 4:**
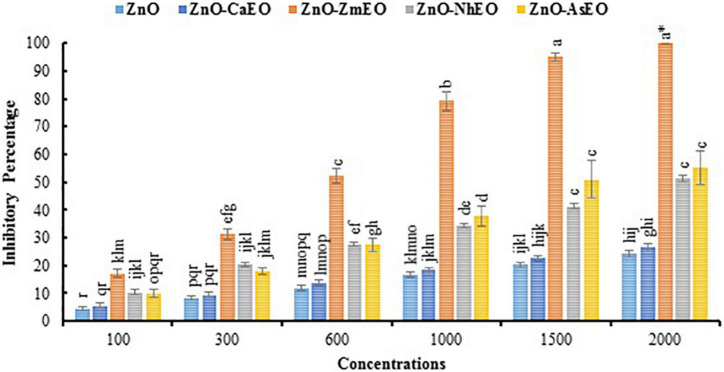
Comparison of mean inhibitory percentage of *A. solani* mycelial growth with ZnO and nanocapsules of essential oils *in vitro*. The symbol “*” indicates the fungistatic property.

Macroscopic images of the mycelial growth inhibitory (MGI) effect of EOs tested on *A. solani* are shown in Figure 2. It can be found that as the concentration of *Z. multiflora* EO and ZnO-ZmEO increased, the rate of *A. solani* growth inhibition was augmented (Figures 2A,B). Growth inhibition percentages caused by concentrations of 1,500 and 2,000 ppm from the pure *Z. multiflora* EO were placed in different statistical groups in accordance with Tukey’s test (*p* < 0.05), but there was no significant difference in nanocapsulated formulation. Both *Z. multiflora* EO and its nanocapsule caused 100% growth inhibition percentage and had a fungistatic effect on the seventh day (Figures 3, 4). The results of Probit analysis showed that the calculated IP_50_ values for *Z. multiflora* EO and ZnO-ZmEO were 471.6 and 323.03 ppm, respectively. In other words, nanocapsulated EO had high antifungal activity due to lower IP_50_ (Table 4).

**TABLE 4:** Probit analysis of data obtained from the inhibitory effect of samples on *A. solani in vitro*.

Samples	Inhibition percentage 50% (90% fiducially limit)	Chi-square (df = 4)	Intercept	Slop	Significant
ZnO	19917.1 (6703.5–344948.8)	0.35	−2.79	0.65	0.98
ZnO-CaEO	21833.25 (6818.1–550123.7)	0.72	−2.59	0.59	0.94
ZnO-ZmEO	323.036 (92.4–606.9)	39.05	−4.97	1.98	0.00
ZnO-NhEO	1764.251 (1367.3–2520.1)	1.08	−3.80	1.17	0.89
ZnO-AsEO	1866.674 (1463.2–2621.1)	0.69	−4.17	1.27	0.95
*Citrus aurantifolia*	18994.8 (7120.3–201525.2)	3.82	−3.82	0.815	0.43
*Zataria multiflora*	471.6 (232.5–773.9)	38.23	−7.42	2.77	0.00
*Nepeta haussknechtii*	2768.1 (2033.6–4442.9)	1.59	−4.28	1.24	0.81
*Artemisia sieberi*	6865.845 (3649.2–24167.8)	4.87	−3.21	0.83	0.97

Macroscopic images of the mycelial growth inhibition of *A. solani* affected by *N. haussknechtii* EO and ZnO-NhEO are illustrated in Figures 2C,D. The time of exposure and the tested concentrations of *N. haussknechtii* EO and ZnO-NhEO had significant inhibitory effects on the mycelial growth effect of *A. solani* (Figures 3, 4). The concentration of 2,000 ppm of ZnO-NhEO caused a 51.25% inhibitory effect on *A. solani* mycelial growth rate. Based on the results of Probit analysis, the IP_50_ values of *N. haussknechtii* EO and ZnO-NhEO were calculated as 2,768 and 1,764 ppm, respectively.

Macroscopic images of the mycelial growth inhibition of *A. sieberi* affected by *A. sieberi* EO and ZnO-AsEO are shown in Figures 2E,F. The exposure times and concentrations of *A. sieberi* EO and ZnO-AsEO had also significant inhibitory effects on the mycelial growth effect of *A. solani* (Figures 3, 4). Calculated IP_50_ values for the pure EO and nanocapsulated formulations were 6,865 and 1,866 ppm after 7 days, respectively.

Macroscopic images of the mycelial growth inhibition of *A. sieberi* treated by *C. aurantifolia* EO and ZnO-CaEO are presented in Figures 2G,H. The exposure times and concentrations of *C. aurantifolia* EO and ZnO-CaEO had also significant inhibitory effects on *A. solani* (Figures 3, 4). IP_50_ values for *C. aurantifolia* EO and ZnO-CaEO were 18,994 and 21,833 ppm after 7 days, respectively. Hence, it is noticed that the antifungal effects of *C. aurantifolia* EO and ZnO-CaEO on *A. solani* were lower than the other tested materials (Table 4).

As seen, the ZnO-ZmEO material has the highest effect on fungal growth. The pure *Z. multiflora* EO at the lowest concentration (100 μl/L) was comparable with the ZnO and ZnO-ZmEO materials on the fungal growth. Therefore, by employing a small concentration of EO, it is possible to remarkably improve the effect of ZnO NP. In the ZnO-ZmEO, the inhibition of mycelial growth was enhanced to 74.22% compared to the ZnO. In comparison with the pure *Z. multiflora* EO, inhibition by ZnO-ZmEO has also been improved to 53.81%. The fungal growth inhibition rate in *Z. multiflora* EO was 44.20% higher than in pure ZnO (Figures 3, 4).

### *In vivo* Antifungal Study

**FIGURE 5:**
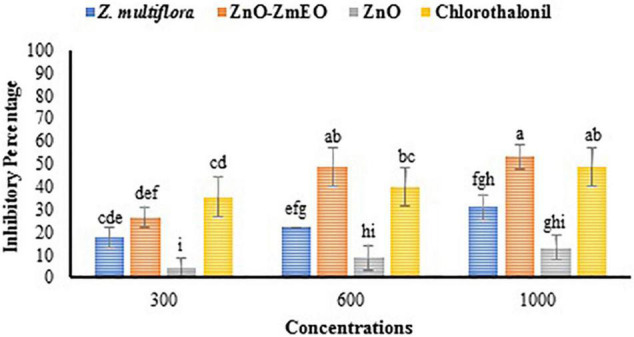
Comparison of mean inhibitory percentage of ZnO, ZnO-ZmEO, pure *Z. multiflora* EO, and chlorothalonil on early blight disease.

**FIGURE 6:**
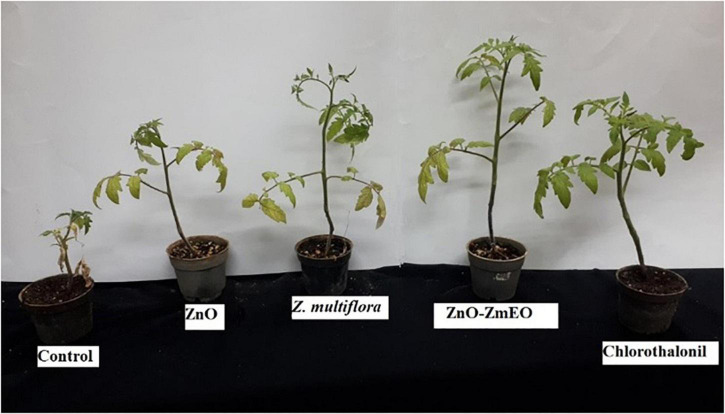
*In vivo* antifungal activity of the ZnO, pure *Z. multiflora* EO, ZnO-ZmEO, and chlorothalonil against *Alternaria solani* on tomato plants.

Different concentrations of *Z. multiflora* EO, ZnO, and ZnO-ZmEO had diverse effects on the early blight disease on tomato plants. The disease symptoms were diminished with increasing tested agents’ concentrations so that 13.33, 31.11, and 53.33% of symptoms were reduced by the highest concentration (1,000 ppm) of ZnO, *Z. multiflora* EO, and ZnO-ZmEO, respectively. Plant samples treated with chlorothalonil at the same concentration showed a 48.89% reduction in disease symptoms (Figure 5). The ZnO-ZmEO had more than 75 and 41.66% inhibition than ZnO and pure EO, respectively (Figure 6). Based on the results of ANOVA, the antifungal effect of ZnO-ZmEO was significant at the tested concentrations (*F* = 5.17, df = 2, *p* = 0.036), but *Z. multiflora* EO and ZnO had no significant effect by concentrations used. The IP_50_ value for ZnO-ZmEO was 771.8 ppm, while it was 4,337.3 and 9,615.9 ppm for *Z. multiflora* EO and pure ZnO, respectively.

**FIGURE 7:**
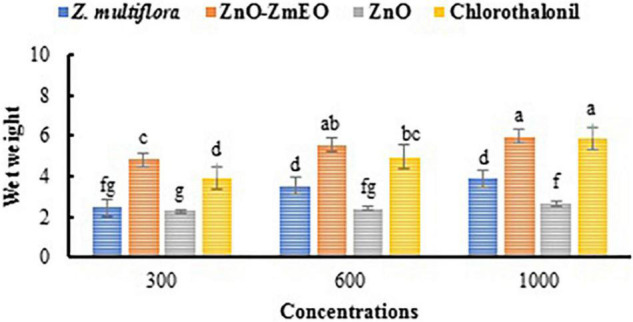
Comparison of mean wet weight of tomato plants treated by different concentrations of the ZnO, pure *Z. multiflora* EO, ZnO-ZmEO, and chlorothalonil.

**FIGURE 8:**
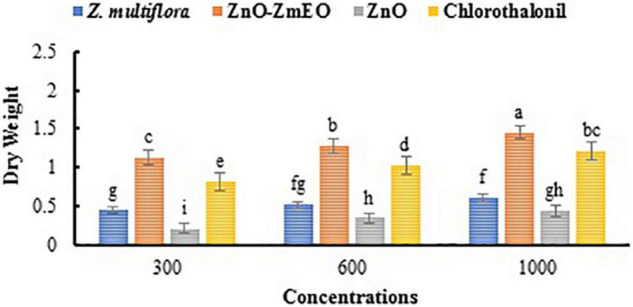
Comparison of mean dry weight of tomato plants treated by different concentrations of the ZnO, pure *Z. multiflora* EO, ZnO-ZmEO, and chlorothalonil.

As phytotoxicity is a probable side effect of the application of botanical agents (Kaveh et al., 2014), high concentrations of *Z. multiflora* EO and ZnO-ZmEO were not tested in this study. The highest concentration used in this study was 1,000 ppm, which had more than 50% inhibition on phytopathogenic fungus and had not any phytotoxicity. The highest wet weight belonged to ZnO-ZmEO-treated plants (5.95 g), which was not statistically different from chlorothalonyl treatments (5.87 g) based on the comparison of the mean (*p* < 0.05). Also, the lowest wet weight of tomato plant was observed in ZnO (2.62 g) (Figure 7). Also, the highest dry weight was related to ZnO-ZmEO-treated plants (1.45 g), which was not statistically different from chlorothalonyl-treated plants (1.21 g) based on the comparison of the mean (*p* < 0.05). Also, the lowest dry weight of tomato plant was observed in ZnO (0.43 g) (Figure 8).

## Discussion

Thymol, γ-terpinene, *p*-cymene, and carvacrol were recognized as the main components of *Z. multiflora* EO, in agreement with the findings of Mahboubi et al. (2017) and Farahpour et al. (2021). However, there are quantitative differences in identified compounds. For example, the percentage of thymol in this study was 47.08%, while its corresponding amount in the Mahboubi et al. (2017) and Farahpour et al. (2021) studies was 25.80 and 52.9%, respectively. The ability of the thymol is to change the hyphal morphology and cause hyphal aggregates, resulting in reduced diameters and lyses of hyphal wall. Also, thymol is lipophilic, enabling it to interact with the cell membrane of fungus cells, altering cell membrane permeability by permitting the loss of macromolecules (Moghtader, 2012). The major constituents of *A. sieberi* EO are β-thujone, 1,8-cineole, camphor, germacrene D, and terpinen-4-ol. In the study by Aati et al. (2020), although camphor (24.1%) was the main compound, similar to our findings, other compounds such as β-thujone, 1,8-cineole, camphor, germacrene D, and terpinen-4-ol were not recognized. Limonene, α-terpineol, and γ-terpinene are the major compounds in *C. aurantifolia* EO, which were detected in the study by Song et al. (2022) with different percentage. Limonene has a variety of functions such as defense against predators and pathogens, as well as a signal to harmless organisms as pollinators for plants. Additionally, it has been reported that limonene affects the signaling pathway and cell membrane. Limonene also inhibits the growth of yeast fungi by damaging cell walls and plasma membranes. However, many ingredients in EO such as γ-terpinene and α-terpineol in addition to limonene have antifungal activity and can affect the antifungal activity of lime (Ehsanfar et al., 2020). Jamzad et al. (2008) indicated that 1,8-cineole, elemol, thymol, germacrene D, and γ-terpinene were the main compounds in *N. haussknechtii* EO. Although 1,8-cineole and germacrene D were also recognized in this study, there was no trace from elemol and γ-terpinene in *N. haussknechtii* EO. Accordingly, there are notable differences in EO compositions investigated in present and previous studies. In fact, several factors, including environmental conditions, geographical origin, genetic make-up, and even phonological stages of plants and agricultural practices, can affect the chemical composition of EOs, resulting in the abovementioned differences (Moghaddam and Mehdizadeh, 2017).

It was found that the biological effects of plant EOs are associated with their chemical compounds, and the antifungal efficiency of some identified compounds in this study was approved (Kazemi et al., 2011; Dehghani Bidgoli, 2021; Karimi and Meiners, 2021). For example, 1,8-cineole, as one of the major constituents of EOs of *N. haussknechtii* and *A. sieberi* EOs, has been shown to inhibit the growth of pathogenic fungi (Garg and Siddiqui, 1992), which may be attributed to its capability to damage cell membrane (Varshney et al., 2012). According to the study by Dehghani Bidgoli (2021), antifungal activity of *Artemisia* genus is due to the presence of the biological secondary metabolites such as bornyl acetate, camphor, and 1,8-cineol. Karimi and Meiners (2021) revealed that *Z. multiflora* EO comprises the effective phenolic compounds such as carvacrol and thymol, causing high antifungal activities on a wide range of major food and agricultural pathogens. Furthermore, it was found that thymol causes substantial morphological damage to the microbial cell membrane, changing the permeability and release of cellular contents (Di Pasqua et al., 2006; Moosavy et al., 2008). Therefore, the stronger antifungal properties of *Z. multiflora* EO than other ones can be due to the presence of active metabolites such as thymol, which was not found in others.

In this research, the inhibitory effect of ZnO on the mycelial growth of *A. solani* was investigated. The outcomes manifested that raising the concentration was useful in inhibiting the fungus mycelial growth. He et al. (2011) showed that a high concentration of ZnO possessed a good inhibitory effect on *B. cinerea* and *Penicillium expansum* Link. The antimicrobial (Kim et al., 2012) effects of nanoparticles on bacteria, fungi, and viruses have been well studied. The antibacterial performances of ZnO and silver nanomaterials have recently been demonstrated (Kumar et al., 2008). Due to the remarkable combination of superior physicochemical, chemical, optical, and considerable environmental stability, cost-effective, and non-toxic properties, ZnO has attracted much interest as one of the multifunctional metal oxide nanoparticles (Salahuddin et al., 2015). In addition to the booster effect related to zinc act as a precursor in cytokinins, auxins, and gibberellins biosynthesis, and the induction of activity of antioxidant enzymes against the pathogenic attacks, ZnO NPs have promising antifungal effects and can be applied for controlling *F. oxysporum* in tomatoes (González-Merino et al., 2021).

Based on previous research, metal nanoparticles are absorbed by the leaves in the atmosphere around the plant, and it has been proven that the structure of stoma and hairs is affected by this nanoparticle (Silva et al., 2006). Various studies have shown that ZnO is non-toxic to human cells and toxic to bacterial cells, and as a result, these nanoparticles are compatible with human cells (Sirelkhatim et al., 2015). The results of Mosquera-Sánchez et al. (2020) represented that the synthesized ZnO-NPs are suitable, cost-effective, and valuable antifungal alternatives to be employed in agricultural production systems, particularly in the protection of crops. Two mechanisms can be used to protect plants using nanoparticles; in the first, nanoparticles protect the plant directly, and in the second, nanoparticles act as carriers with different advantages such as high durability, improving the solubility of water-soluble poisons and reduce toxicity (Hayles et al., 2017).

Over the last decade or so, nanoencapsulation and microencapsulation technologies have been explored for their efficiency to improve the handling, dispersibility, and stability of hydrophobic substances, as well as to control their release profiles (Weisany et al., 2022). In this study, ZnO-ZmEO had the highest inhibition of growth *in vitro* compared to other nanocapsules of EOs and pure *Z. multiflora* EO, so it was compared with the chemical fungicide in the greenhouse conditions and showed a significant reduction in symptoms. Previous research has shown that nano-EO formulation has the power of gradual release and increases the durability of EOs and nanocapsulated EOs have an antifungal activity higher than the pure EO and ZnO (Emamjomeh et al., 2018). The results of Upadhyay et al. (2021) showed that nanocapsules of *Cananga odorata* (Lam.) Hook.f. & Thomson EO completely inhibited the growth of *Aspergillus flavus* and demonstrated an enhanced antioxidant activity. In Enayati et al.’s (2021) study, to obtain an effective alternative for highly toxic synthetic chemicals, the antifungal properties of *Z. multiflora* EO loaded on ZnO material were assessed against six isolates of *Fusarium*. The findings demonstrated that ZnO-EO nanocomposite possessed an antifungal effect on all investigated fungi except *F. oxysporum* f.sp. *lentis* and the fungicidal activity toward *F. graminearum* at a concentration of 1,000 ppm.

## Conclusion

Essential oils have numerous potential applications in the agriculture, food, and pharmaceutical industries. However, their hydrophobicity, chemical instability, and volatility pose a challenge for many of their applications. One of the best approaches to solve these challenges is to encapsulate the EOs in nanosize or encapsulation EOs in colloidal delivery systems (Enayati et al., 2021; Weisany et al., 2022). In this study, nanocapsules of some EOs were successfully prepared and ZnO-*Z. multiflora* EO possessed the best performance compared to other EOs, nanocapsules of other EOs and ZnO *in vitro* because of its smaller particle size and higher distribution surface. Then, this study highlighted the advantage of nanocapsules over the common form of EOs like *Z. multiflora* EOs. Also, ZmEO was used with its nanocapsules and ZnO *in vivo* with concentrations that showed better inhibition in the laboratory, and the results illustrated that ZnO-ZmEO has the highest percentage of inhibition (53% reduction) in the control of tomato early blight disease in greenhouses and after consideration of additional research can be used as an alternative for high-risk chemical fungicides that are harmful to health and the environment.

## Data Availability Statement

The original contributions presented in this study are included in the article/supplementary material, further inquiries can be directed to the corresponding author.

## Author Contributions

AA performed the experiments. MD and AH-Y supervised the experiments from the beginning to the end and read and corrected the manuscript. AE advised the research, made statistical analyses, analyzed the EOs, and participated in proof-writing and editing the final version of manuscript. SF advised the research and performed the nanoparticle synthesis. All authors contributed to the article and approved the submitted version.

## Conflict of Interest

The authors declare that the research was conducted in the absence of any commercial or financial relationships that could be construed as a potential conflict of interest.

## Publisher’s Note

All claims expressed in this article are solely those of the authors and do not necessarily represent those of their affiliated organizations, or those of the publisher, the editors and the reviewers. Any product that may be evaluated in this article, or claim that may be made by its manufacturer, is not guaranteed or endorsed by the publisher.
